# Isoflurane Anesthesia Initiated at the Onset of Reperfusion Attenuates Oxidative and Hypoxic-Ischemic Brain Injury

**DOI:** 10.1371/journal.pone.0120456

**Published:** 2015-03-23

**Authors:** Sergey A. Sosunov, Xavier Ameer, Zoya V. Niatsetskaya, Irina Utkina-Sosunova, Veniamin I. Ratner, Vadim S. Ten

**Affiliations:** Department of Pediatrics, Division of Neonatology, Columbia University, New York, New York, United States of America; Albany Medical College, UNITED STATES

## Abstract

This study demonstrates that in mice subjected to hypoxia-ischemia (HI) brain injury isoflurane anesthesia initiated upon reperfusion limits a release of mitochondrial oxidative radicals by inhibiting a recovery of complex-I dependent mitochondrial respiration. This significantly attenuates an oxidative stress and reduces the extent of HI brain injury. Neonatal mice were subjected to HI, and at the initiation of reperfusion were exposed to isoflurane with or without mechanical ventilation. At the end of HI and isoflurane exposure cerebral mitochondrial respiration, H_2_O_2_ emission rates were measured followed by an assessment of cerebral oxidative damage and infarct volumes. At 8 weeks after HI navigational memory and brain atrophy were assessed. In vitro, direct effect of isoflurane on mitochondrial H_2_O_2_ emission was compared to that of complex-I inhibitor, rotenone. Compared to controls, 15 minutes of isoflurane anesthesia inhibited recovery of the compex I-dependent mitochondrial respiration and decreased H_2_O_2_ production in mitochondria supported with succinate. This was associated with reduced oxidative brain injury, superior navigational memory and decreased cerebral atrophy compared to the vehicle-treated HI-mice. Extended isoflurane anesthesia was associated with sluggish recovery of cerebral blood flow (CBF) and the neuroprotection was lost. However, when isoflurane anesthesia was supported with mechanical ventilation the CBF recovery improved, the event associated with further reduction of infarct volume compared to HI-mice exposed to isoflurane without respiratory support. Thus, in neonatal mice brief isoflurane anesthesia initiated at the onset of reperfusion limits mitochondrial release of oxidative radicals and attenuates an oxidative stress. This novel mechanism contributes to neuroprotective action of isoflurane. The use of mechanical ventilation during isoflurane anesthesia counterbalances negative effect of isoflurane anesthesia on recovery of cerebral circulation which potentiates protection against reperfusion injury.

## Introduction

Neonatal hypoxia-ischemia (HI) brain injury remains one of the major causes of life-long neurological morbidity. Although, permanent brain injury occurs due to severe oxygen and nutrient deprivation, the mechanisms of post-ischemic damage are initiated by reperfusion. Oxidative stress is one of these mechanisms where mitochondria play a central role in generation of injurious reactive oxygen species (ROS). Upon reintroduction to oxygen mitochondria quickly reactivate electron transport in their respiratory chains [1], an event vital for cellular survival. However, the same process accelerates a release of deleterious ROS from mitochondria [2]. Our group and others have shown that inhibition of complex-I (C-I) during ischemia and/or reperfusion significantly reduced the extent of ischemic damage to the developing and mature brains and hearts. This was associated with attenuation of oxidative stress [3–5]. However, agents (pyridaben or rotenone) used for inhibition of C-I in experimental ischemia-reperfusion injury are not translatable for clinical practice.

Isoflurane anesthesia used as pre-or post-treatment protects immature brains against HI-injury [6–9]. Because isoflurane inhibits C-I activity [10, 11], and similarly to a C-I inhibitor, rotenone, reduces ROS generation in mitochondria supported with succinate [11], we hypothesized that these metabolic effects of isoflurane contribute to neuroprotective mechanisms of post-conditioning with isoflurane anesthesia. Almost immediate on/off pharmacological action makes isoflurane an excellent drug of choice for reversible inhibition of C-I recovery in reperfusion in order to reduce a reperfusion-driven surge in ROS production from mitochondria. This study was undertaken to determine whether in the neonatal mouse model of HI-brain injury isoflurane anesthesia initiated at the onset of reperfusion attenuates oxidative damage to HI-brain by inhibition of mitochondrial ROS generation surge.

## Materials and Methods

### The model of unilateral HI brain injury and study groups

**Fig 1:**
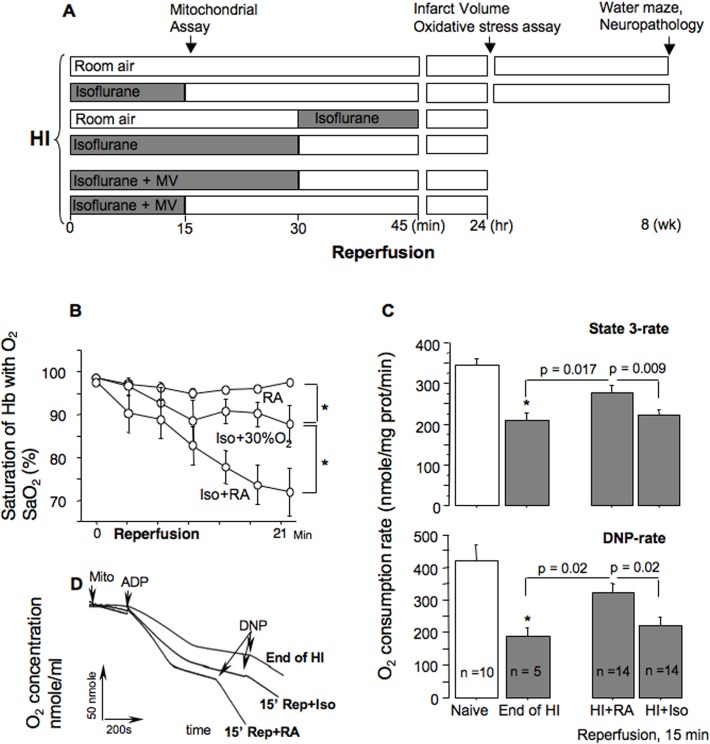
Experimental design. **Mitochondrial phosphorylating respiration rates after HI**. (A)—HI-mice, upon reperfusion, were exposed to either room air or isoflurane with or without mechanical ventilation (MV) for different time of reperfusion. (B)—Changes in SaO_2_ in naive mice (n = 6), and mice exposed to 2 Vol% isoflurane with (n = 6) or without (n = 4) 30% oxygen supplementation, * p < 0.01. (C)—Mitochondrial phosphorylating and uncoupled respiration rates in naïve mice (n = 10), HI-mice at the end of HI-insult (n = 5), and at 15 minutes of reperfusion under isoflurane (HI+Iso, n = 14) or without (HI+RA, n = 14) isoflurane anesthesia. (D)—Representative cerebral mitochondrial respiration tracings from HI-mice tested at the end of HI (End of HI) and 15 minutes of reperfusion with isoflurane exposure (15’ Rep+Iso) or room air (15’ Rep+RA).

All studies were approved by the Columbia University Institutional Animal use Committee according to AAALAC standards. We used the Rice-Vannucci model of regional HI-brain injury in rats [12], adapted to p10 C57Bl6/J neonatal mice [3, 13]. The model consisted of a ligation of the right common carotid artery followed by recovery for 1.5 h and hypoxic (8% O_2_ balanced N_2_) exposure for 15 minutes at the ambient temperature 37.0–37.5°C, as we described [3, 13]. Following hypoxic exposure one group of HI-mice was re-oxygenated with room air (RA)—HI-control. Other three groups of mice were re-oxygenated with 0.9 MAC (2.0 Vol%) isoflurane for either 15 or 30 minutes of initial reperfusion, or for 15 minutes starting at 30 minutes of reperfusion (Fig. 1A). Isoflurane exposure was supplemented with 30% oxygen in order to maintain systemic SaO_2_ at physiological range ≥ 90% (Fig. 1B). The dose of isoflurane (0.9 MAC or 2 Vol%) for p10 C57Bl6/J mice was selected according to the report [14]. In this report authors concluded that similar to human neonates, isoflurane anesthesia in neonatal mice should be combined with mechanical ventilation (MV). Therefore, a separate cohort of mice was exposed to post-HI 0.9 MAC isoflurane anesthesia combined with MV. In this experiment MV was performed as we described [15] with minor modifications. In brief, immediately following HI-insult mice were exposed to isoflurane, their trachea was intubated with angiocath 24G and mice were placed on the respirator MicroVent 848 (Harvard Apparatus). The tidal volume was 6 μl/g of body weight. The respiration rate was kept at 110 breath/minute to maintain paCO_2_ within physiological range. The duration of isoflurane exposure was 15 or 30 minutes of initial reperfusion (Fig. 1A). Once isoflurane exposure was discontinued, mice were extubated and returned to their dams. To determine the extent of brain injury in spontaneously respiring animals, 136 mice were used, and 44 mechanically-ventilated mice were used. For a long-term neurological assessment 49 mice were used. These mice were sacrificed by cervical dislocation under isoflurane anesthesia at 24 hours following HI-insult. In addition, 90 neonatal mice were used for mitochondrial and oxidative brain damage assay. These mice were sacrificed at 0 and 15 minutes and 24 hours of reperfusion by cervical dislocation.

### The measurement of SaO_2_, cerebral blood flow and blood gases

Systemic saturation of the circulating arterialized hemoglobin with oxygen (SaO_2_) was measured in a separate cohort of naïve p10 mice subjected to 0.9 MAC isoflurane with or without oxygen supplementation and compared to naïve animals kept in room air. To measure SaO_2_, a pulseoxymetry probe was placed around the neck and SaO_2_ values were constantly recorded up to 30 minutes using mouse pulseoximeter (Starr Life Sciences, Pittsburgh, PA). In randomly selected HI-animals changes in cerebral blood flow (CBF) in response to HI and re-oxygenation with or without 0.9 MAC isoflurane and ventilation were measured using laser Doppler flowmeter “Periflux 5000”, as we described [1, 16]. Briefly, under isoflurane anesthesia a laser-Doppler probe was attached to the ipsilateral hemisphere skull using 15 cm long fiberoptic extension. Fiberoptic extension was placed 3 mm lateral to and 2 mm posterior to the bregma. CBF value was expressed as % of the initial pre-hypoxic level. In a separate group of naïve p10 mice exposed to 0.9 MAC isoflurane for 15 minutes with or without MV blood gases analysis was performed using EPOC analyzer (Epocal, Alere Inc.) in samples obtained from the right common carotid artery.

### Short- and long-term assessment of the HI-brain injury

At 24 hours of reperfusion mice were sacrificed, brains were harvested, sectioned into 1 mm thick coronal slices and stained with 2% triphenyl-tetrazolium chloride (TTC). Digital images of infracted and viable areas of brains were traced (Adobe Photoshop 4.0.1) and analyzed (NIH image 1.62J) by the investigator (S.S.) “blinded” to a study groups. The extent of brain injury was expressed as a percentage of the hemisphere ipsilateral to the carotid artery ligation side.

A long-term neurological assessment was performed at 8 weeks after HI by evaluation of navigational memory in the Morris water maze. The test was carried out as we described [13], with minor modifications. For three consecutive days (three trials a day) mice were trained to find a submerged platform in the pool Ø110 cm. On the day four, mice were offered a probe trial, when a platform was removed and time spent in the “platform” quadrant was recorded. The allotted time on probe trial was 60 seconds. At the completion of navigational memory evaluation mice were euthanized. Brains were removed and fixed in 4% paraformaldehyde. Coronal sections (40 μm thick every 500 μm) were Nissl stained. Digital images were obtained and processed as described above. The extent of cerebral atrophy in the ipsilateral hemisphere was defined as percentage in relation to corresponding contralateral hemisphere (100%).

### Assessment of mitochondrial function

At 0 and 15 minutes of reperfusion cerebral non-synaptic mitochondria were isolated from the ipsilateral hemisphere as we described [3, 17]. In brief, brain hemisphere was harvested and immediately immersed into 2 ml of ice-cold isolation buffer. The tissue was homogenized manually using a dounce homogenizer (Wheaton Ind., NJ) with 0.2 mm differential (10 strokes) and followed by 0.1 mm differential (10 strokes). The homogenate was centrifuged at 1100 g for 2 min in a refrigerated (+4°C) table-top centrifuge (Eppendorf 5810R). The pellets were discarded and the 0.750 ml of supernatant was mixed with 0.07 ml of 80 vol% Percoll solution, carefully layered on top of 0.7 ml of 10% Percoll solution and centrifuged at 18,500 g for 10 min. The mitochondria—enriched fraction was collected at the bottom of the tube and re-suspended in 1.0 ml of sucrose washing buffer. The suspension was centrifuged at 10,000 g for 5 min. The final mitochondrial pellet was re-suspended in 0.07 ml of albumin-free washing buffer, and stored on ice. Using NAD or FAD-linked substrates, malate-glutamate or succinate, respectively, mitochondrial respiration and H_2_O_2_ emission rates were measured. To assess direct effect of isoflurane on mitochondrial H_2_O_2_ generation rate, cerebral mitochondria isolated from naïve p10 mice were exposed to the buffer pre-bubbled (60 seconds) with either RA (normoxia), or 100% O_2_ with or without 2 Vol% isoflurane. In vitro hyperoxia imitates a post-ischemic re-oxygenation. Rotenone (stock 0.5 mM in 96% alcohol, 2 μl / 1000 μl buffer) was used as a control for inhibition of C-I. Alcohol (96%, 2 μl / 1000 μl buffer) was used as a vehicle.

Mitochondrial respiration was measured using a Clark-type electrode (Oxytherm, Hansatech, UK), as we described [1, 13, 16]. Briefly, mitochondria (0.05 mg of protein) were added to 0.5 ml of respiration buffer: 10 mM MOPS-Tris, pH 7.4, 120 mM KCl, KH_2_PO_4_ 1 mM, EGTA 10 μM, 0.2 mg/ml of BSA, 30 μM Ap_5_A (*P* ^1^,*P* ^5^-di(adenosine 5')-pentaphosphate), 10 mM glutamate, and 5 mM malate at t = 32°C. Phosphorylating respiration was initiated with 100 nmol of ADP and uncoupled respiration was initiated by adding 40μM of 2’3 dinitrophenol (DNP).

Mitochondrial H_2_ O_2_ emission was estimated by Amplex ultrared fluorescence assay using Hitachi 7000 spectrofluorimeter set at 555 nm excitation and 581 nm emission as described earlier [3, 18]. Briefly, mitochondria (0.05 mg/ml) were placed in 1 ml of respiratory buffer containing 5 mM succinate or 5mM malate/10 mM glutamate, 10 μM amplex ultrared and 4 U/ml of horse radish peroxidase (HRP) and the rate of H_2_O_2_ fluorescence raise was recorded. At the end of each experiment five aliquots of 100 nmol of H_2_O_2_ was added to the respiration buffer every 60 sec to obtain a calibration curve. The rates of H_2_O_2_ emission were expressed in pmolH_2_O_2_/mg of protein/min.

### Assessment of oxidative injury to the brain tissue and mitochondria

Oxidative damage to mitochondria (decreased aconitase activity) was assessed as we described [3]. Frozen-thawed mitochondria were mixed with the reaction buffer (50 mmol/L Tris-HCl, pH 7.4, 0.6mmol/L MnSO4, 5mmol/L Na citrate, 0.5 mmol/L nicotinamide adenine dinucleotide phosphate (NADP), 1 U/mL iso-citrate dehydrogenase) in a 96-well plate and the absorbance changes at 340nm were followed for 10 minutes with a plate reader (Tecan Infinite M200, San Jose, CA, USA). The aconitase activity was expressed in mU per minute per mg of mitochondrial protein. Aconitase activity was expressed in mU/min/mg of mitochondrial protein. Oxidative proteins damage in the brain was evaluated by detection of 3-nitrotyrosine (3-NT) using western blot (anti-3NT, 1:1000; Millipore).

### Statistical analysis

One-way ANOVA with Fisher’s post-hoc analysis was used to detect statistical differences in brain infarct volumes, navigational memory and mitochondrial functions. Changes in CBF and SaO_2_ were analyzed using ANOVA for repeated measures with Fisher’s post-hoc analysis. T-test was used to detect differences in the brain infarct volume between HI-mice exposed to isoflurane with or without mechanical ventilation. All data are mean ± SEM. Difference was considered statistically significant if p-value < 0.05.

## Results

### Post-HI isoflurane anesthesia inhibits recovery of C-I dependent respiration and the reperfusion-driven acceleration in mitochondrial ROS release

**Fig 2:**
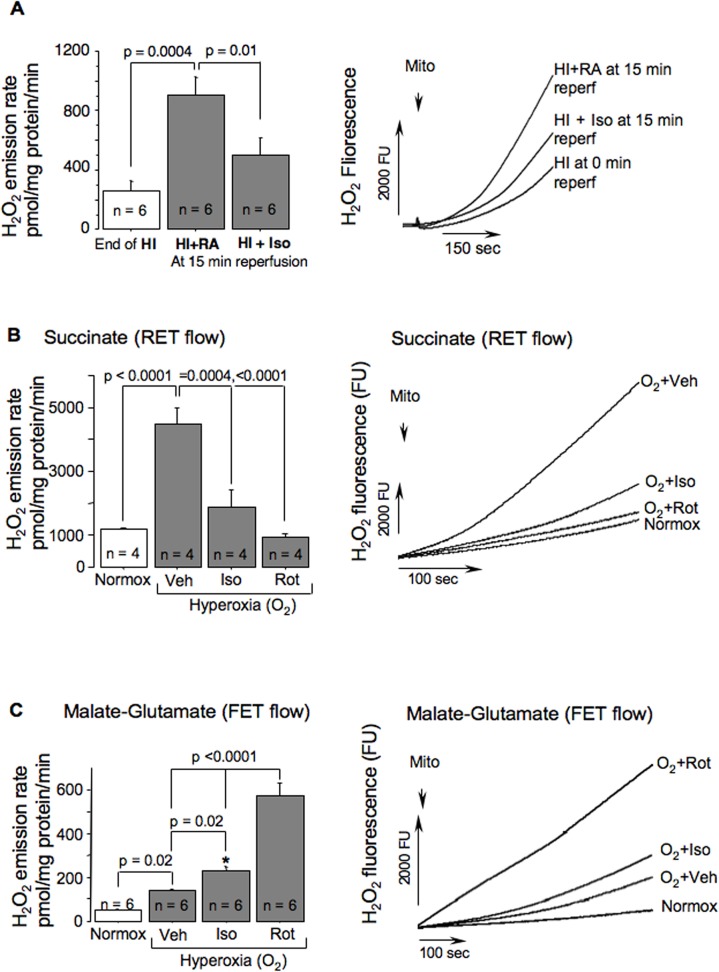
In-vitro and ex-vivo effect of isoflurane on mitochondrial ROS release. (A)—Mitochondrial H_2_O_2_ emission rate and representative H_2_O_2_ fluorescence tracings in HI-mice at the end of HI (n = 6) and at 15 minutes of reperfusion with (n = 6) or without (n = 6) isoflurane anesthesia. (B and C)—H_2_O_2_ emission rates with representative H_2_O_2_ fluorescence tracings from mitochondria fueled with succinate (B) or malate-glutamate (C) and exposed to hyperoxic buffer (O_2_) in the presence of vehicle (O_2_ + Veh, n = 4 and 6), or Isoflurane (O_2_ + Iso, n = 4 and 6), or Rotenone (O_2_ + Rot, n = 4 and 6) and compared to controls (Normox, n = 4 and 6). P-values and study groups are indicated. * p < 0.01 compared to normoxia.

Compared to mitochondria isolated from naïve mice, mitochondria isolated from the hypoxic-ischemic hemisphere at the end of HI-insult, before isoflurane exposure, exhibited significant depression of C-I dependent phosphorylating and DNP-accelerated respirations (Fig. 1C and D). At 15 minutes of reperfusion in mice reoxygenated with RA mitochondrial respiration recovered compared to that measured at the end of HI. In contrast, in mice reoxygenated under isoflurane anesthesia, cerebral mitochondria continued to exhibit depressed C-I dependent state 3 and uncoupled respirations (Fig. 1C and D). When the same mitochondria from the mice reoxygenated with RA were fueled with C-II dependent substrate, succinate, we found a brisk acceleration of H_2_O_2_ emission compared to that measured at the end of HI (Fig. 2A). However, exposure to isoflurane significantly attenuated this acceleration of succinate-supported H_2_O_2_ emission from post-HI mitochondria (Fig. 2A).

### Isoflurane inhibits mitochondrial H_2_O_2_ emission surge induced by hyperoxia

These *in vitro* experiments imitate a surge in mitochondrial ROS production initiated by the reintroduction of O_2_ into ischemic brain. In response to increase in oxygen content cerebral mitochondria fueled with succinate exhibited a dramatic (p < 0.0001) acceleration of the H_2_O_2_ emission rate compared to normoxia (Fig. 2B). However, in the presence of isoflurane, this hyperoxia-induced surge in mitochondrial H_2_O_2_ production was significantly blunted (Fig. 2B). Similar effect was achieved by an inhibition of C-I with rotenone (Fig. 2B). When the same organelles were supported with malate-glutamate, the baseline H_2_O_2_ emission rate was very low, ∼ 30 folds slower compared to the succinate-fueled H_2_O_2_ production. Hyperoxia significantly increased ROS emission rate (Fig. 2C), especially in the presence of rotenone and to a lesser extent, in the presence of isoflurane (Fig. 2C).

### Post-HI isoflurane anesthesia is associated with reduced oxidative injury

**Fig 3:**
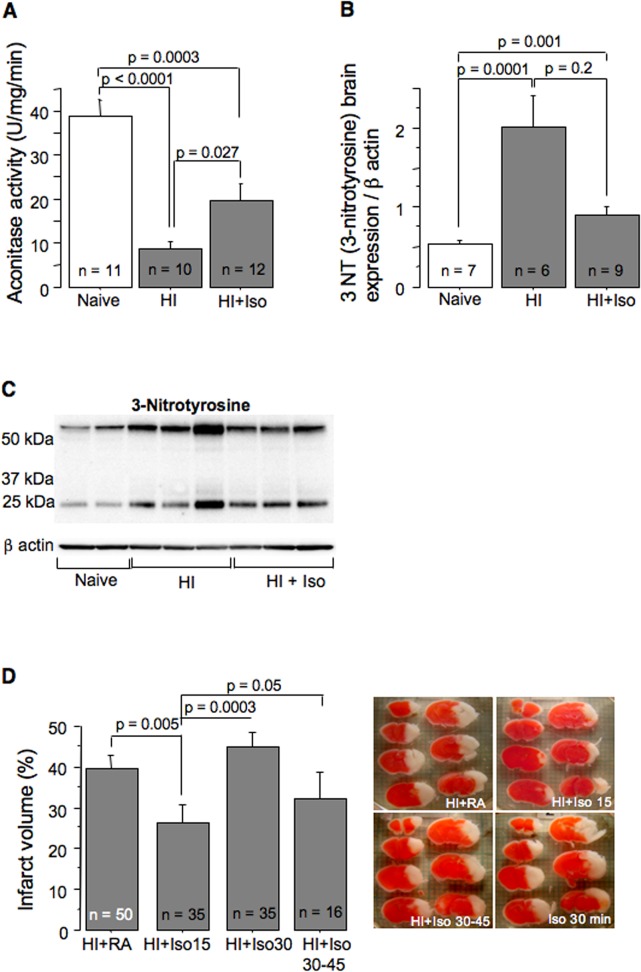
Post-HI isoflurane anesthesia attenuates oxidative brain damage and extent of brain injury. (A and B)—Mitochondrial aconitase activity and expression of 3-Nitrotyrosine in brains obtained from naïve mice (n = 11 and 7) and in HI-mice reperfused for initial 15 minutes with isoflurane (n = 12 and 9) or RA (n = 10 and 6). (C)—representative 3-Nitrotyrosine western blot. (D)—Infarct volume and representative TTC-stained cerebral images of HI-mice reperfused without (RA, n = 50), or with isoflurane anesthesia: for initial 15 min (n = 35), or initial 30 min (n = 35), or delayed (30–45 minutes, n = 16).

Compared to naïves, the aconitase activity, a marker of oxidative damage to the mitochondrial matrix, was significantly decreased in HI-mice reoxygenated with RA or isoflurane (Fig. 3 A). However, compared to RA-reoxygenated HI-mice, HI-mice exposed to isoflurane exhibited significantly greater preserved aconitase activity (Fig. 3 A). A marker of protein oxidative damage, 3NT, was significantly increased following HI-insult (Fig. 3 B and C). In the HI-mice treated with isoflurane, 3NT-level was significantly lower compared to the RA-treated HI-mice (Fig. 3 B and C).

### Only brief isoflurane anesthesia initiated after HI is neuroprotective

**Fig 4:**
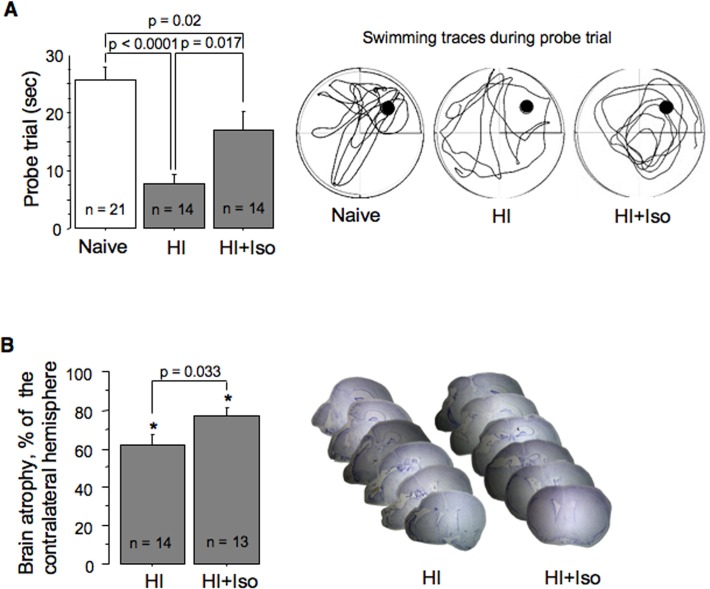
Long-term neurological outcome of the HI-brain injury after isoflurane exposure. (A)—Navigational memory: time spent in the “platform quadrant” by naïve mice (n = 21) and HI-mice re-oxygenated without (HI, n = 14), or with isoflurane (HI+Iso, n = 14). Representative tracings of swimming path during probe trial in the same groups of mice. (B)—Extent of brain atrophy in the ipsilateral hemisphere and representative Nissl-stained brain images from adult mice treated with isoflurane for initial 15 minutes of reperfusion (HI+Iso, n = 13) or RA (HI, n = 14).

At 24 hours of reperfusion HI-mice exposed to isoflurane for initial 15 minutes of reperfusion demonstrated significantly decreased cerebral infarct volume compared to their RA reoxygenated controls (Fig. 3D). However, when the same isoflurane exposure was delayed and was initiated at 30 minutes of reperfusion no neuroprotection was observed (Fig. 3D). Surprisingly, an extension of post-HI isoflurane anesthesia to 30 minutes of initial reperfusion also did not affect the extent of brain injury compared to RA reoxygenated controls (Fig. 3D). In these mice the infarct volume was significantly greater compared to that in HI-mice post-treated with the same regimen of isoflurane for 15 minutes (Fig. 3D). Thus, the neuroprotection was detected only in the HI-mice post-treated with isoflurane for initial 15 minutes. Therefore, this group of mice was studied for a long-term neurological outcome. At 10 weeks of life these mice exhibited significantly better navigational memory and significantly better-preserved ipsilateral hemisphere compared to their RA-reoxygenated controls (Fig. 4A, B).

### Isoflurane anesthesia alters post-HI recovery of the cerebral blood flow in spontaneously breathing mice

**Fig 5:**
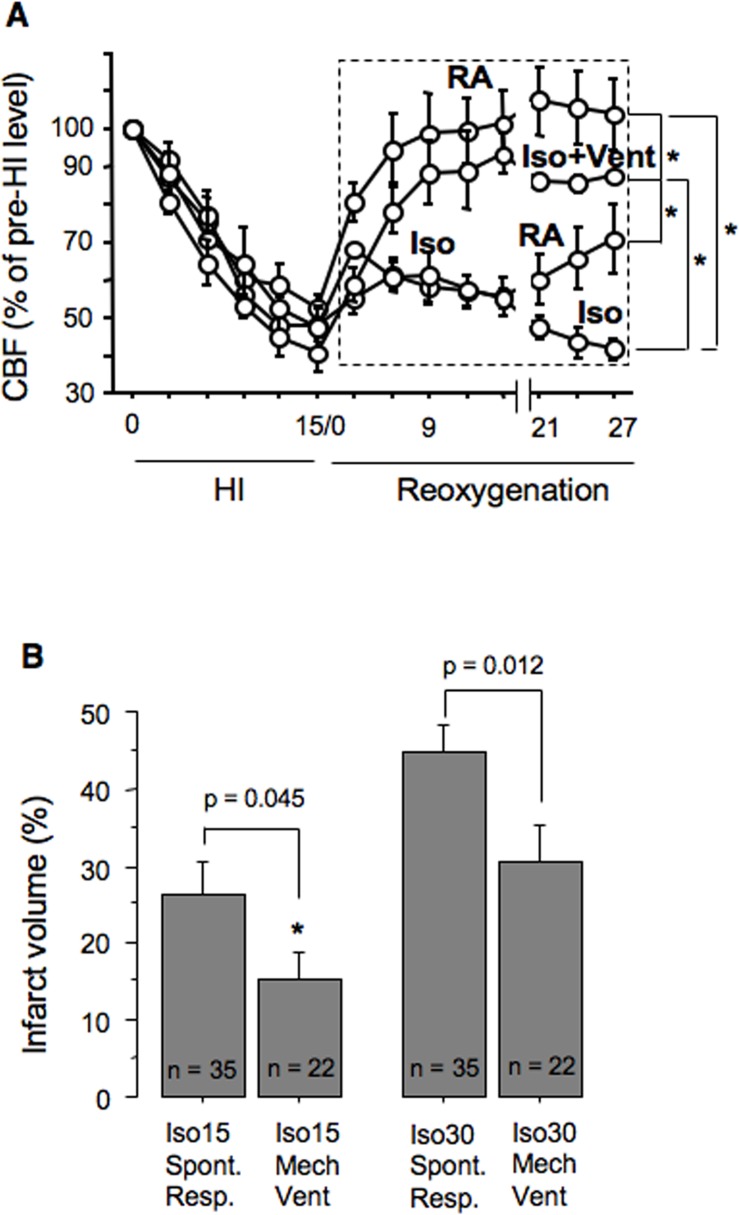
Mechanical ventilation enhances neuroprotection of isoflurane. (A)—CBF during HI and reperfusion in mice re-oxygenated with RA (n = 4) or Isoflurane for initial 15 minutes (Iso—RA, n = 4), or 30 minutes (Iso, n = 4), or mice re-oxygenated with isoflurane combined with mechanical ventilation (Iso+Vent, n = 4). * p < 0.02 between groups, Dashed square indicates analyzed area. (B)—Infarct volume in mice re-oxygenated under isoflurane anesthesia for 15 or 30 minutes with (n = 22) or without (n = 35) mechanical ventilation. * p = 0.017 compared to the mice ventilated for 30 minutes.

Compared to the control HI-mice, their littermates anesthetized with isoflurane without MV exhibited significantly poorer recovery of the CBF (Fig. 5A). In mice exposed to isoflurane for 15 minutes a brisk CBF recovery was detected only when anesthesia has been discontinued (Fig. 5A). If isoflurane anesthesia was extended, the CBF remained depressed (Fig. 5A). Compared to naïve animals, isoflurane exposure in spontaneously breathing mice significantly increased paCO_2_ (Table 1). The use of MV during isoflurane anesthesia normalized paCO_2_, HCO_3_^−^ and significantly improved CBF recovery (Fig. 5A).

**Table 1:** Blood gases during isoflurane anesthesia with or without MV.

Mice	pH	paCO2	HCO3-
Naïve n = 5	7.41 ± 0.025	38.6 ± 3.5	24.6 ± 0.98
Iso. n = 4	7.25 ± 0.031	69.0 ± 5.03*	30.7 ± 0.39*
Iso. MV n = 4	7.33 ± 0.01	44.0 ± 4.00	23.2 ± 1.7

* p < 0.002 compared to Iso. MV group. Data are Mean ± SEM

### Mechanical ventilation during post-HI isoflurane anesthesia enhances neuroprotective action of isoflurane

Because in spontaneously breathing HI-mice isoflurane anesthesia depressed cerebral reperfusion, we reasoned that this sluggish CBF recovery counterbalances beneficial metabolic effect of isoflurane. Indeed, compared to spontaneously respiring HI-mice, the exposure to isoflurane with MV significantly greater reduced infarct volume (Fig. 5B). The use of MV with 30 minutes of isoflurane anesthesia also significantly decreased the extent of brain damage compared to that in spontaneously breathing counterparts (Fig. 5B). However, no neuroprotection was achieved compared to the vehicle-treated HI-mice (mean ± SE infarct volumes: vehicle = 39.7 ± 2.78 vs Isoflurane = 30.4 ± 5.07, p = 0.08).

## Discussion

This report is the first to demonstrate that in neonatal HI-mice, post-conditioning with isoflurane anesthesia, initiated at the onset of reperfusion, inhibited recovery of C-I dependent mitochondrial respiration and significantly reduced a reperfusion-driven surge in generation of mitochondrial ROS. This significantly reduced an oxidative injury to the HI-brain, the event associated with permanent neuroprotection.

Post-ischemic mitochondria are one of the major sources of oxidative radicals released during reperfusion. The mechanism of ROS generation in mitochondrial respiratory chain depends on mitochondrial substrate which defines directions of electron transport flow, affecting sites and rates of ROS generation. In organelles fuelled with succinate, a reverse electron transport (RET) flow (electron flux proceeds backward from C-II, ubiquinone to C-I and to the matrix-located NAD) serves as a primary mechanism for generation of ROS. The main site of ROS production in RET flow is C-I [19]. Here and in other studies we have found a brisk acceleration of ROS production in response to reperfusion in mitochondria fueled with succinate (Fig. 2A) [3]. This has been attributed to a rapid recovery of RET-flow in C-I. Because C-I inhibition, for example with rotenone, interrupts RET-flow and dramatically decreases ROS emission [2, 18], it has been proposed that an inhibition of C-I dependent recovery of RET-flow reduces formation of ROS in the post-ischemic brain and heart [3, 20]. Our study demonstrates that in vitro effect of isoflurane on RET and FET flows-dependent mitochondrial H_2_O_2_ generation fully simulated the action of C-I inhibitor, rotenone. Therefore, we propose that in our vivo experiment isoflurane limits RET-driven mitochondrial ROS generation upon reperfusion. Similar results were obtained in isolated heart ischemia-reperfusion injury pre-exposed to isoflurane [20]. Our study supports a pathogenic role of the ROS originating in C-I during RET-flow by demonstrating a novel action of isoflurane anesthesia; attenuation of oxidative injury secondary to inhibition of the recovery of C-I. Thus, in the post-HI brain isoflurane inhibits reactivation of C-I which limits a surge of ROS release during restoration of RET-flow in reperfusion. This data-interpretation agrees with report showing reduced accumulation of hydroxyl radicals and products of lipid peroxidation achieved by an inhibition of C-I with rotenone or haloperidol in rats with cerebral ischemia [4]. Inhibition of C-I with amytal also reduced level of free radicals and lipid peroxidation in isolated rabbit and rat hearts subjected to ischemia-reperfusion [5, 21], and similar effect of isoflurane on isolated cardiac mitochondria has been reported [22].

Our data on mitochondrial ROS emission were obtained using ex-vivo isolated organelles fuelled with succinate. The rationale for the use of succinate rests on a body of evidence suggesting, that succinate serves as a primary mitochondrial substrate at the onset and early reperfusion in the ischemic hearts [23, 24] and brains [25, 26]. In the rat brain, ischemia profoundly depleted all NAD-linked substrates, but concentration of succinate was increased by ∼ 300% [25], and remained elevated for 15 minutes of reperfusion [25, 26]. In neonatal asphyxiated piglets concentration of circulating succinate in reperfusion reached 8000% (!) of the pre-asphyxia level [27]. Finally in rats, brain ischemia significantly inhibited C-I dependent mitochondrial respiration, but when the same mitochondria were tested on succinate, no difference from control values was detected [28]. Importantly, succinate supports the highest rate of ROS generation in brain mitochondria [19, 29]. Indeed, we demonstrate a dramatic (4.5 folds) increase in succinate-supported mitochondrial ROS generation in the in vitro environment, mimicking reintroduction of O_2_ during reperfusion. In contrast, in the same organelles fueled with glutamate-malate a considerable rise in ROS emission was achieved only when C-I was inhibited with rotenone or isoflurane. This suggests that unless C-I is inhibited, potential contribution of NAD-linked respiration to ROS production is very modest compared to that of FAD-linked respiration. Just recently, Chouchani et al demonstrated that in the heart, brain and other organs the level of mitochondrial succinate dramatically (3–20 folds) increased during ischemia, and succinate-supported mitochondrial respiration was responsible for accelerated mitochondrial ROS generation during reperfusion [36]. Similarly to the earlier reports [25, 26], these authors also demonstrated a rapid (5 min) decrease of cerebral succinate to the pre-ischemic level during reperfusion [36]. The latter suggests a return of mitochondrial preference in substrate oxidation from succinate to NAD-linked substrates. This also suggests that in the later stages of reperfusion, proposed strategy with inhibition of C-I may become ineffective, as inhibited C-I in the mitochondria oxidizing NAD-linked substrates may contribute to elevated mitochondrial ROS emission. This may explain a loss of neuroprotection with delayed or prolonged use of isoflurane in this study. Thus, in neonatal HI-mice temporal therapeutic window of isoflurane post-treatment is restricted to the onset and very early (initial 15 minutes) stage of reperfusion which supports metabolic link of isoflurane neuroprotection to a post-ischemic mitochondrial preference of succinate oxidation.

The loss of neuroprotection with an extention of isoflurane anesthesia from 15 minutes to 30 minutes was unexpected finding, and this result is very intriguing. In spontaneously respiring mice the absence of protection with prolonged isoflurane post-treatment can be explained by the inhibiting effect of isoflurane on CBF recovery which limits normalization of cerebral O_2_ content. These data are consonant with reports that Isoflurane anesthesia alters autoregulation of regional and global cerebral blood flows [30, 31]. When post-HI isoflurane anesthesia was supported with MV, the recovery of CBF has improved. This significantly augmented the neuroprotective effect of isoflurane in mice exposed to isoflurane for 15 minutes, and decreased infarct volume in mice anesthetized for 30 minutes. However, only mice exposed to isoflurane for 15 minutes of the initial reperfusion demonstrated significantly reduced infarct volume compared to vehicle-treated animals. MV and isoflurane exposure for 30 minutes resulted in a strong tendency (p = 0.08) toward neuroprotection compared to spontaneously respiring vehicle treated animals. Thus, the use of MV is very important in experimental research of neuroprotective properties of general anesthetics. It has been reported that isoflurane anesthesia, in the dose similar to that used in our study, was lethal in spontaneously respiring p10 mice when isoflurane was applied during HI-insult. The use of MV, however, significantly improved survival [14]. Importantly, even with the use of MV, isoflurane anesthesia exhibited a short therapeutic window, because extended (30 minutes) use of isoflurane with MV did not exert neuroprotection. These data, at first glance, seem to be in a conflict with reports showing that a prolongation of isoflurane pre-treatment from 4 to 25 minutes or an increase of the dose have reduced the extent of HI brain injury in neonatal rats [32, 33]. However, in contrast to above referenced reports, in our study we have used a post-treatment paradigm. Therefore, potential mechanisms of anti-ischemic action of isoflurane used as the pre-conditioning differs from that of the post-conditioning used here. It is also important to note, that anti-NMDA receptor and GABA-type A receptor-stimulating action of isoflurane were proposed as neuroprotective mechanisms in the pre- or intra-treatment settings, when the exposure to isoflurane was carried-out prior- to or during oxygen-glucose deprivation [34, 35]. Our work does not contradict to above referenced studies, but shows another mechanism when the anesthetic is used at the initiation of reperfusion. This mechanism contributes to known mechanisms of isoflurane neuroprotection,

In conclusion, as a post-treatment strategy, only brief isoflurane anesthesia initiated at the onset of reperfusion significantly reduced brain injury, providing permanent neurological benefit in neonatal HI-mice. The mechanism for this neuroprotection is related to deceleration of mitochondrial ROS production during reperfusion and attenuation of oxidative injury. However, temporal window of this therapeutic effect in neonatal mouse model of HI is short and restricted to the initial reperfusion stage. Nevertheless, this finding does not cross out a translational significance of our study, as in human neonates the timing of post-HI metabolic shift favoring neuroprotective effect of isoflurane may differ from that in neonatal mice. Therefore, further studies with the use of non-rodent animal models of HI should be focused on the reperfusion timing when mitochondria—substrate interaction is optimal for neuroprotective action of isoflurane.

## Supporting Information

S1 Dataset Mitochondrial respiration data. (XLSX)

S2 Dataset Cerebral infarct volume data. (XLSX)
